# Preoperative carbohydrate loading reduces length of stay after major elective, non-cardiac surgery when compared to fasting: a systematic review and meta-analysis

**DOI:** 10.1038/s41598-025-00767-z

**Published:** 2025-05-31

**Authors:** Anna Réka Sebestyén, Caner Turan, Ambrus Szemere, Marcell Virág, Klementina Ocskay, Fanni Dembrovszky, László Szabó, Péter Hegyi, Marie Anne Engh, Zsolt Molnár

**Affiliations:** 1 Centre for Translational Medicine, Semmelweis University, Budapest, 1085 Üllői út 26 Hungary; 2 Heim Pál National Pediatric Institute, Budapest, Hungary; 3 Department of Anaesthesiology and Intensive Therapy, Semmelweis University, Budapest, Hungary; 4 Szent György University Teaching Hospital of Fejér County, Székesfehérvár, Budapest, Hungary; 5 Institute for Translational Medicine, Medical School, University of Pécs, Pécs, Hungary; 6 Institute of Pancreatic Diseases, Semmelweis University, Budapest, Hungary; 7 Department of Anesthesiology and Intensive Therapy, Faculty of Medicine, Poznan University of Medical Sciences, Poznan, Poland

**Keywords:** Preoperative fasting, Oral carbohydrate loading, Major surgery, Non-cardiac surgery, Length of stay, Diseases, Medical research

## Abstract

Preoperative fasting is a worldwide routine even though the most recent Enhanced Recovery After Surgery (ERAS) Guidelines recommend preoperative carbohydrate loading instead of fasting, but with low quality of evidence. Our aim was to compare the effects of preoperative carbohydrate loading to fasting and placebo in patients undergoing elective major non-cardiac surgery under general anaesthesia. Our systematic search was conducted on 15th of October 2021 in five databases, Medline, Embase, Central, Web of Science and Scopus, and updated on November 12th, 2024. We included randomized controlled trials that compared the carbohydrate loading (CHO-group) with fasting or with placebo. Main outcomes were length of hospital stay (LOS), postoperative glucose levels on postoperative, postoperative insulin levels, and C-reactive protein (CRP) levels. Our search revealed 44 eligible articles for data extraction. LOS was shorter in the CHO group as compared to the No-CHO group (MD: − 0.56 [95% CI: − 1.10, − 0.02]). There were no clinically significant differences between the CHO and No-CHO groups regarding the postoperative glucose, insulin and CRP levels. This meta-analysis found that preoperative CHO-loading as compared to preoperative fasting or placebo shortened the length of hospital stay in patients undergoing major elective, non-cardiac surgery. Although several details are still to be unveiled, these data provide further support that preoperative carbohydrate loading could be beneficial in this patient population.

**Supplementary Information:**

The online version contains supplementary material available at 10.1038/s41598-025-00767-z.

## Introduction

Preoperative fasting, when patients are forbidden to consume food and drink after midnight prior to surgery, is still a common practice worldwide^1^. The reason behind this strategy is to reduce the risk of aspiration of stomach contents during induction of and emergence from anaesthesia^2^.

Despite the increasing evidence that fasting may increase the risk of postoperative complications, such as metabolic stress manifesting in hyperglycemia, insulin resistance and a more pronounced inflammatory response, preoperative fasting remains a common practice^3^. However, the most recent Enhanced Recovery After Surgery (ERAS) Guidelines recommend the intake of solid food 5–6 h before surgery, clear fluids up to 2 h and even strongly recommend preoperative carbohydrate loading (CHO)^4^.

Carbohydrate loading may have several potential benefits such as less metabolic stress, and less pronounced inflammatory response and shorter hospital stay, however, clear evidence is still lacking^5^.

Although the recommendation is strong, the quality of the evidence remains low or moderate in the majority of the guidelines^4^. Therefore, our aim was to synthetize and re-analyze all relevant literature to assist policymakers and guideline authors by generating the highest level of evidence in decision making.

## Methods

We report our systematic review and meta-analysis based on the recommendations of the PRISMA 2020 guideline^6^, while we followed the Cochrane Handbook^7^. The protocol of the study was registered on PROSPERO (CRD42021284663) and we fully adhered to it.

### Eligibility criteria

Randomized controlled trials (RCTs) were included, reporting on patients undergoing elective major non-cardiac surgery under general anesthesia. In accordance with Cochrane Handbook’s recommendations for meta-analyses of interventions, non-randomized clinical studies, case series, case reports, editorials, commentaries, and other qualitative studies were excluded. Articles available only in abstract form or meeting reports were also excluded. We applied no filters, no restrictions on methodological quality, publication date or language. Major surgery was defined as any invasive operative procedure in which a more extensive resection is performed, e.g. a body cavity is entered, organs are removed, or normal anatomy is altered. Non-cardiac surgery was defined as every type of major surgery except cardiac. We defined preoperative carbohydrate loading (CHO) as a carbohydrate, mineral and vitamin containing amount of fluid which is consumed maximum 2 h prior surgery (CHO-group). Those studies that utilized more interventions of the ERAS guideline were excluded to investigate the effect of carbohydrates only. Preoperative fasting was defined as no food consuming after midnight prior surgery, which means no breakfast was allowed even when the surgeries were scheduled in the afternoon, making some of the patients fast for 12–15 h. We defined placebo as a fluid, with or without artificial sweeteners, vitamins, and minerals.

### Information sources

Our systematic search was conducted on 15th of October 2021 in five databases, Medline (via Pudmed), Embase, Cochrane Central Register for Controlled Trials (CENTRAL), Web of Science and Scopus.

The systematic search was repeated on the 12th of November 2024 in the same databases.

### Search strategy

During the systematic search the following search key was used: (preoperative AND (‘carbohydrate’/exp OR carbohydrate) OR ‘fasting abbreviation’) AND random* in Embase. In the other four databases, we used (preoperative AND carbohydrate) OR “fasting abbreviation”) AND random*. The same search key was used in the renewed systematic search without any filters, for maximal reproducibility.

### Selection process

The selection was performed by two independent authors (AS and ASz using Endnote X20; CT and MAE for the renewed search), disagreements were resolved by consensus. After automatic and manual duplicate removal, we screened the records based on title and abstract, and after this step, we screened the remaining records based on full text against the predefined eligibility criteria. In cases when multiple studies used the same patient database for the same outcomes, we used only the study with the largest sample in our analysis.

### Data collection process

The data collection was performed by two independent authors (AS and ASz, then CT and MAE for the renewed investigation). The following data were extracted: first author, the year of publications, study population, study period, country, intervention, fasting and placebo group, number of patients, baseline characteristics, length of hospital stay (LOS), postoperative glucose and insulin levels, postoperative CRP levels.

### Pooling of studies

As studies reported data at different time points, we created a general rule for the pooling of data points. We ran an analysis including the earliest measurement of each paper, one for immediately postoperatively, and one for postoperative day 1. A subgroup analysis was performed for the primary outcome, subgroups consisted of major abdominal interventions (including gastrointestinal, urinary tract and gynecological procedures) versus an “other” group.

### Study risk of bias assessment

We assessed the risk of bias by a Cochrane-recommended bias tool for randomized trials (RoB 2.0) for RCTs^8^. Risk of bias assessment was performed by two independent review authors (CT and MAE) and disagreements resolved by consensus. Quality of evidence evaluation was executed following the guidance of the Grades of Recommendation, Assessment, Development, and Evaluation (GRADE) workgroup. Assessment of the quality of evidence was arranged in tables, that were prepared with the GRADEPro Guideline Development Tool^9^.

### Statistics

Data synthesis was performed by using the methods recommended by the Cochrane Collaboration^10^. For continuous outcomes pooled MDs with their 95% CI were calculated to investigate the differences between the compared arms. Where medians were available with interquartile ranges, we estimated the means and standard deviations with the approach suggested by Shi et al.^11^. The restricted maximum likelihood estimator was used. The random effects model was applied for meta-analyses. If the study number for the given outcome was over five, the Hartung–Knapp adjustment^12,13^ was applied.

Statistical heterogeneity across trials was assessed by means of the Cochrane Q test, and the I2 values, where *p* < 0.1 was considered as statistically significant.

The statistical analyses were carried out by R (R Core Team 2021, v 4 1.1, R Foundation for Statistical Computing, Vienna, Austria)^14^ using the meta Schwarzer G. General package for meta-analysis^15^ and Dmetar^16^ packages. Forest plots were used to visualize the findings of the meta-analytical calculations. All the data generated and analyzed are included in this article and in its Supplementary table.

## Results

### Search and selection

**Fig. 1:**
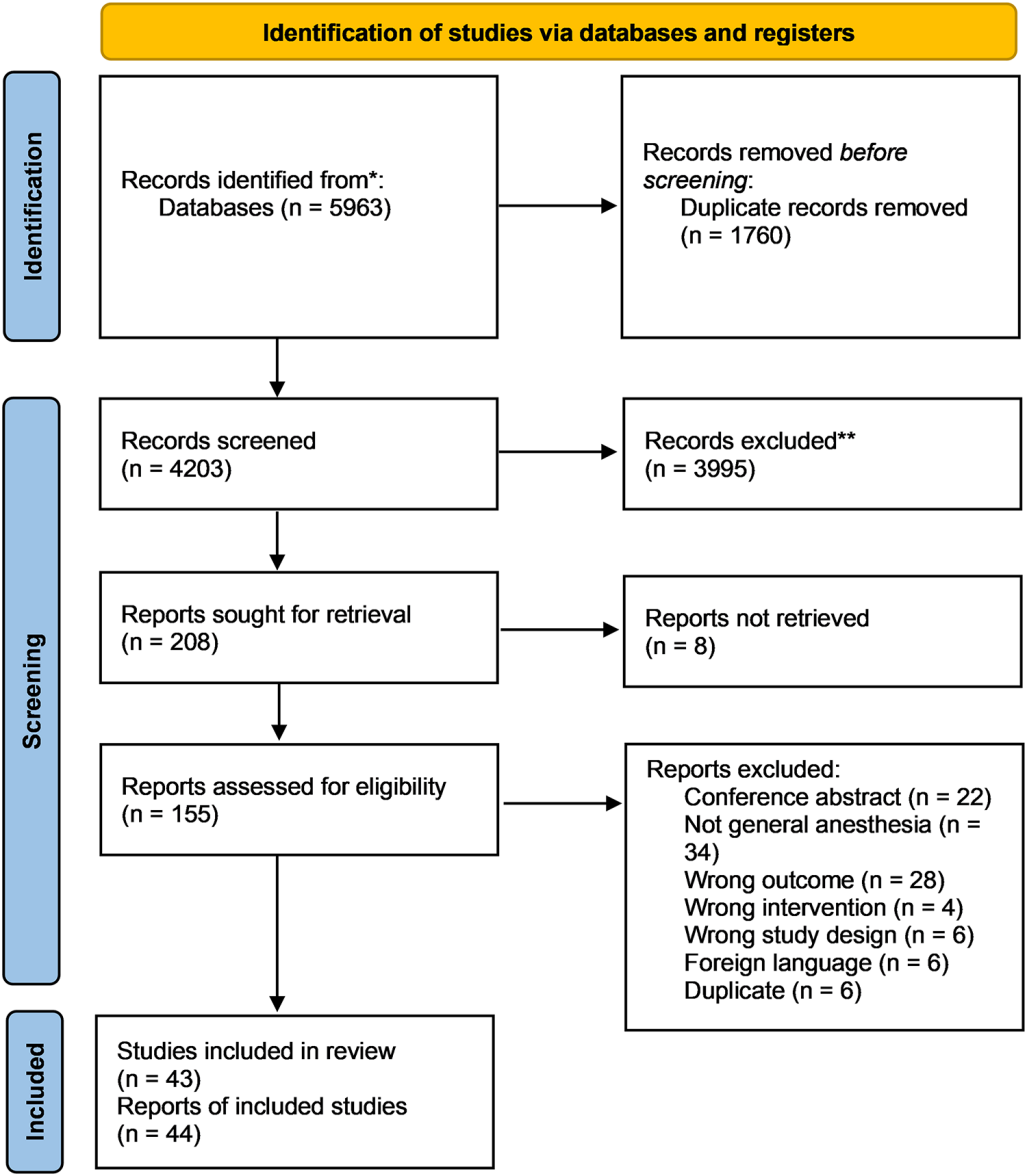
PRISMA flowchart of selection process.

Altogether 5963 studies were identified during our search in Medline (via Pubmed), Embase, Web of Science, Cochrane Central Register of Controlled Trials (CENTRAL) and Scopus databases, and 43 studies^17–57^ were found eligible for inclusion in the meta-analysis and systematic review. The exact details of selection are outlined in the PRISMA flowchart (Fig. 1).

### Basic characteristics of included studies

Baseline characteristics of the enrolled analyses are detailed in Table 1, detailed information on patients in Supplementary Table 2.

**Table 1:** Characteristics of included studies.

Author year	Study period	Overall				Carbohydrate		Comparator
		Country	N0	Type of surgery	Subpopulation			
						Timing	Content	
Cakar 2017	March 2011–September 2011	Turkey	90	Thyroidectomy	No	Midnight, 2 h preop	12.50%	Fasting from midnight
Cho 2021	July 2019 to February 2020	South Korea	64	Laparoscopic benign gynecologic surgery	Female	Midnight, 2 h preop.	12.80%	Fasting from midnight
Cho 2021*	July 2019 to March 2020	South Korea	88	Laparoscopic gynecological surgery	Female	Midnight, 6 a.m., 2 h preop.	12.80%	Fasting from midnight
Deng 2022	March 1, 2017, to February 28, 2018	China	122	Laparoscopic colorectal resection	No	2–3 h before surgery	14.20%	Fasting from midnight
Dilmen 2017	na	Turkey	40	Microsurgical lumbar discectomy	No	Night before, 2 h preop	12.5% oral	Fasting, 8 h
Faria 2009	na	Brazil	21	Laparoscopic cholecystectomy	Female	2 h preop	12.50%	Fasting, 8 h
Gianotti 2018	January 2011–December 2015	Italy	880	Major abdominal surgery (GI, urinary tract, gynecological)	No	2 h preop	12.60%	Water
Gümüs 2021	May 2018 to January 2019	Turkey	68	Laparoscopic cholecystectomy	No	2 h preop	12.50%	Fasting, 12 h
Hamamoto 2018	July 2013 to May 2014	Japan	70	Laparoscopic colon cancer surgery	No	2 h preop	18%	Water
Hausel 2005	na	Sweden	172	Laparoscopic cholecystectomy	No	Night before, 2 h preop	12.50%	Flavored water
Henriksen 2003	na	Denmark	48	Bowel resections	No	Before sleep, 3 h preop	12.50%	Water
Hu 2021	na	China	90	Open radical prostatectomy	Elderly (> 65 years)	2–3 h before surgery	12.60%	Placebo (flavored water with electrolytes)
Kaska 2010	na	Czech Republic	221	Colorectal surgery	No	Evening, up to 2 h preop	12.6	Fasting from midnight
Kumar 2024	March 2021 to February 2023	India	72	Colorectal surgery for cancer	No	10:00, 2 h preop	12.50%	Fasting, 6–8 h
Lai 2024	April 18, 2022, to January 1, 2023	China	na	Primary unilateral total knee arthroplasty	No	2 h preop	12%	Water
Lee 2018	September 2015 to December 2016	South Korea	153	Laparoscopic cholecystectomy	No	Evening before, 2 h preop	12.80%	Flavored water
Li 2022	August 1, 2017 to May 7, 2018	China	63	Major gastrointestinal surgery	T2DM	Evening before, 6 am	14.20%	Fasting
Lin 2022	December 2019 to December 2020	China	78	Not specified	T2DM	2 h preop	14.20%	Fasting
Liu 2019	October 2016 to July 2017	China	120	Elective craniotomy	No	2 h preop	12.5%	Water
Mathur 2023	2022	India	90	Laparoscopic cholecystectomy	No	3 h preop	12.5%	Water
Mathur 2010	July 2004 to December 2005	New Zealand	142	Hepatectomy and colorectal surgery	No	Evening before, then 2 h before surgery	12.5%	Placebo
Noblett 2006	na	UK	36	Colorectal surgery	No	Evening before, then 3 h preop	12.5%	Water or Fasting
Onalan 2019	2016	Turkey	50	Laparoscopic cholecystectomy	No	Evening before, then 2 h preop	12.5%	Fasting
Pedziwiatr 2015	October 2013 to February 2014	Poland	40	Laparoscopic cholecystectomy	No	2 h preop	12.5%	Water
Qin 2022	September 2017 to October 2019	China	231	Gastrointestinal surgeries	No	Evening before, then 2 h preop	12.5%	Water
Rajan 2021	na	India	52	Thyroidectomy	No	2 h preop	15%	Water
Rizvanovic 2019	na	Bosnia and Herzegovina	50	Colorectal surgery	No	2 h preop	12.5%	Fasting
Rizvanovic 2023	May 2020 to January 2022	Bosnia and Herzegovina	60	Colorectal surgery	No	Evening before, then 2 h before surgery	12.5%	Fasting
Sada 2014	January 2010 to January 2012	Kosovo	142	Colorectal surgery or cholecystectomy	No	Evening before, then 2 h before surgery	12.5%	Fasting or placebo
Senapathi 2022	na	Indonesia	68	Elective craniotomy	No	2 h preop	12.5%	Water
Shi 2020	January 2017 to December 2017	China	63	Colorectal surgery	No	2 h preop	12.5%	Fasting or placebo
Sio 2015	March 2014 to February 2016	Korea	142	Colorectal surgery	No	2 h preop	na	Fasting
Tavalaee 2022	June 2020 to December 2020	Iran	95	Laparoscopic cholecystectomy	No	Evening before, then 2 h before surgery	12.5%	Fasting
Varughese 2024	May 2023 to June 2023	India	50	Laparoscopic cholecystectomy	T2DM	5 AM surgery day	12.5%	Placebo
Wang 2019	July 2017 to December 2017	China	73	Endoscopic submucosal dissection	No	Evening before, then 2 h before surgery	5%	Fasting
Wang 2024	December 2022 to June 2023	China	129	Laparoscopic cholecystectomy	No	2 h preop	12.5%	Fasting
Wu 2022	September 2019 to January 2021	China	86	Oromaxillofacial surgery	Elderly	2 h preop	12.5%	Fasting
Yadav 2023	August 2023 to September 2023	India	64	Laparoscopic cholecystectomy	T2DM	2 h preop	12.5%	Fasting
Yang 2012	na	China	60	Radical distal gastrectomy	No	2–3 h preop	10%	Placebo
Yuan 2023	January 2021 to August 2021	China	100	Laparoscopic cholecystectomy	No	2–3 h preop	15.5%	Water
Yuill 2005	August 1999 to March 2001	UK	65	Gastrointestinal surgeries	No	Evening before, then 2–3 h before surgery	12.5%	Placebo
Zhang 2022	na	China	200	Gynecological surgeries	Female	2–3 h preop	na	Fasting

### Length of hospital stay (LOS)

**Fig. 2:**
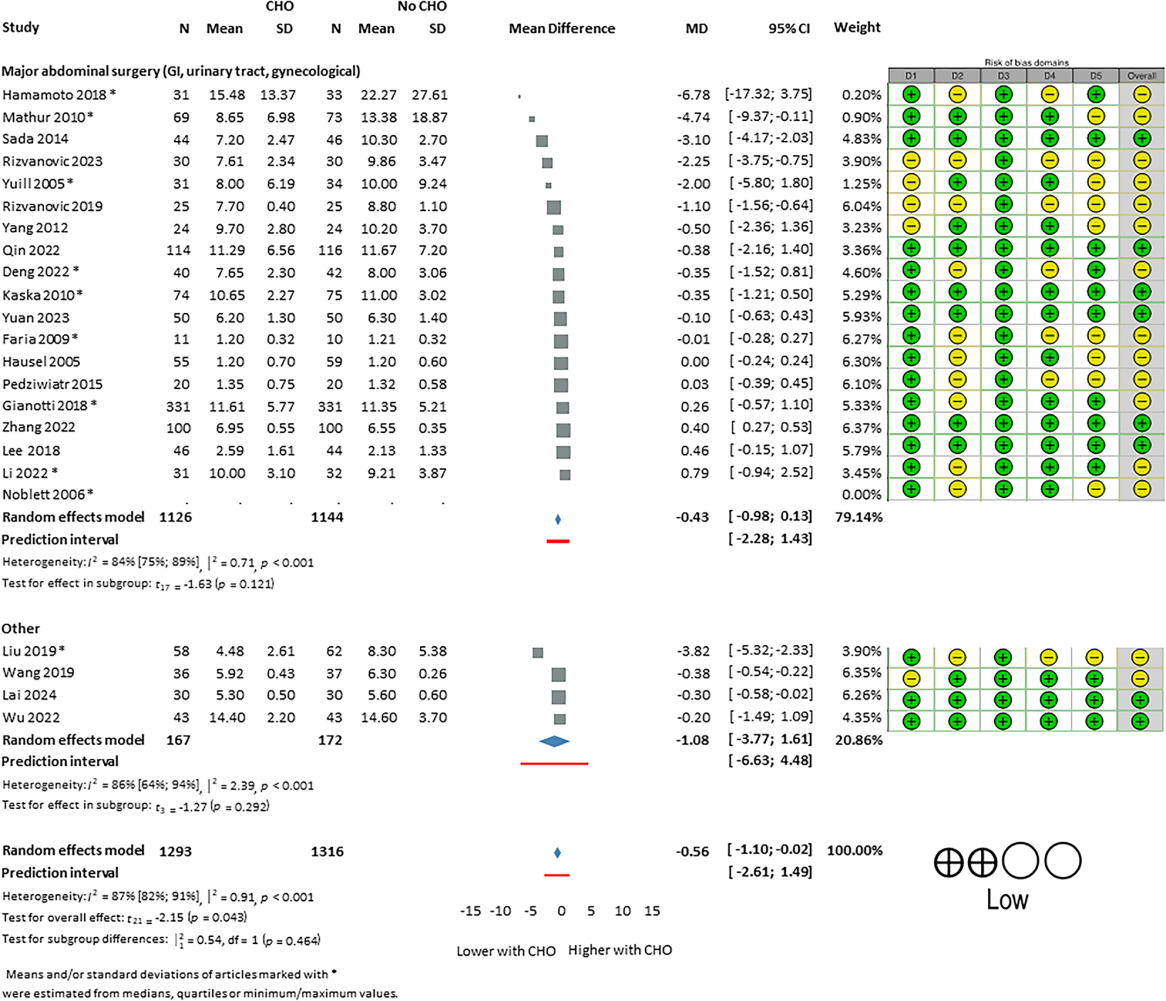
Length of hospital stay (CHO vs. No CHO—fasting or placebo). CHO; preoperative carbohydrate loading. Risk of bias assessment with RoB2 tool displayed next to the respective article where a green (+) refers to low, and a yellow (-) refers to moderate risk of bias. D1: bias arising from randomization process, D2: bias due to deviations from intended interventions, D3: bias due to missing outcome data, D4: bias in measurement of the outcome, D5: bias in selection of the reported result. Summary of the level of certainty of evidence assessment by GRADE assessment on the bottom left corner.

22 studies^20,22,23,25,26,29,31–33,36,37,39,40,42,44,50,53,55,57–59^, including 2609 patients, reported LOS as an outcome. LOS in the CHO-group versus the No-CHO was significantly shorter (MD: − 0.56 [95% CI: − 1.10, − 0.02]) (Fig. 2). Considerable statistical between-study heterogeneity was detected in both cases.

### Postoperative glucose levels

**Fig. 3:**
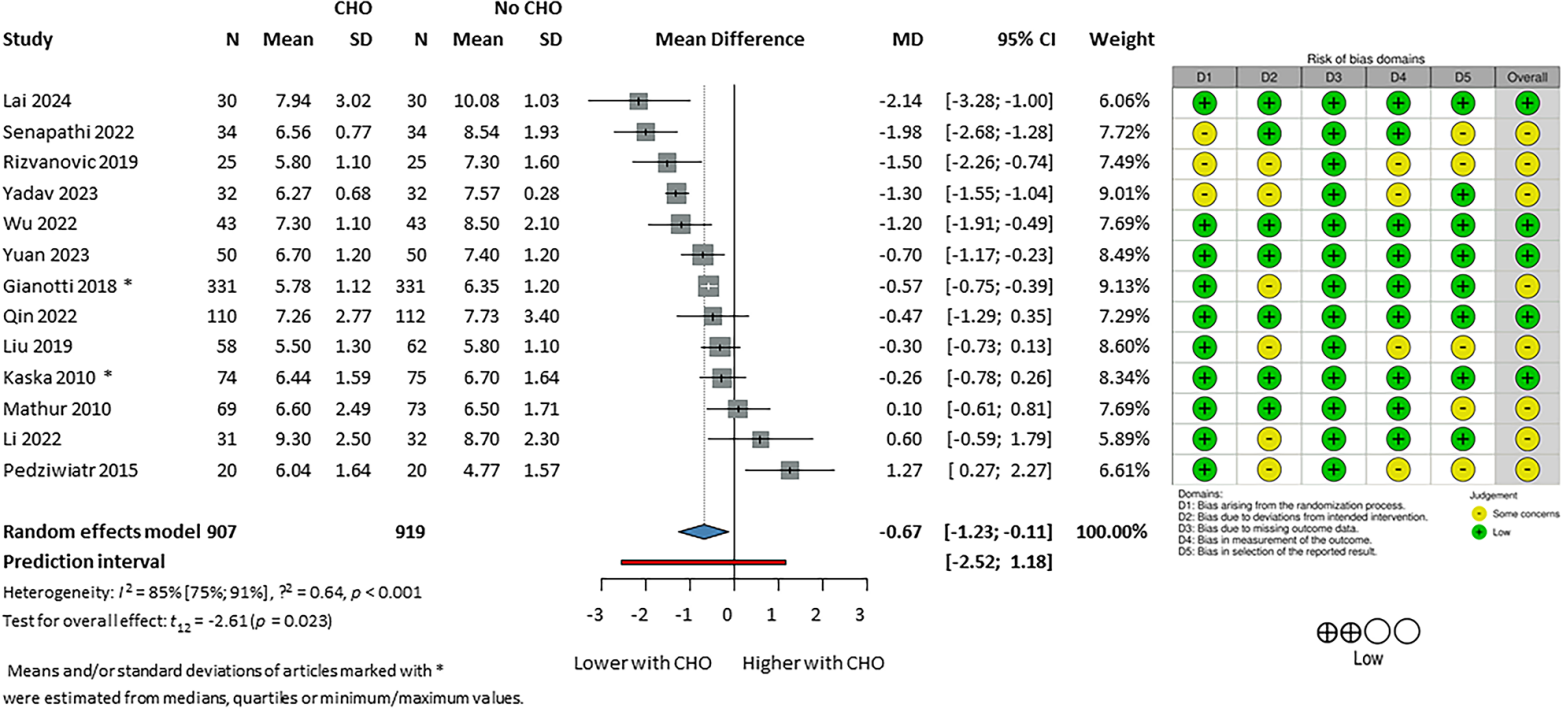
Serum glucose level on Postoperative Day 1 (CHO vs. No CHO—fasting or placebo). CHO; preoperative carbohydrate loading;. Risk of bias assessment with RoB2 tool displayed next to the respective article where a green (+) refers to low, and a yellow (−) refers to moderate risk of bias. D1: bias arising from randomization process, D2: bias due to deviations from intended interventions, D3: bias due to missing outcome data, D4: bias in measurement of the outcome, D5: bias in selection of the reported result. Summary of the level of certainty of evidence assessment by GRADE assessment on the bottom left corner.

13^40,42,45,52,55,59^ studies including 1826 patients reported the serum glucose level on the postoperative day 1 (Fig. 3). These values were pre-prandial and were reported after the first 24-h period postoperatively. The unit of measurement used to report the pooled estimates is mmol/L, and results of studies reported using mg/dL were converted to mmol/L. There was a statistically significant but not clinically important difference between the CHO and No-CHO groups (MD: − 0.67 [95% CI: − 1.23, − 0.11]). Results from the meta-analysis of serum glucose levels measured immediately after the operation, and at the earliest time of measurement in the study are presented at Supplementary Fig. 2 and Supplementary Fig. 3 respectively. Neither show a significant difference between CHO and No-CHO groups: MD: − 0.51 [95% CI: − 1.06, 0.04] and MD: − 0.1 [95% CI: − 0.57, 0.38] respectively).

### Postoperative insulin levels

**Fig. 4:**
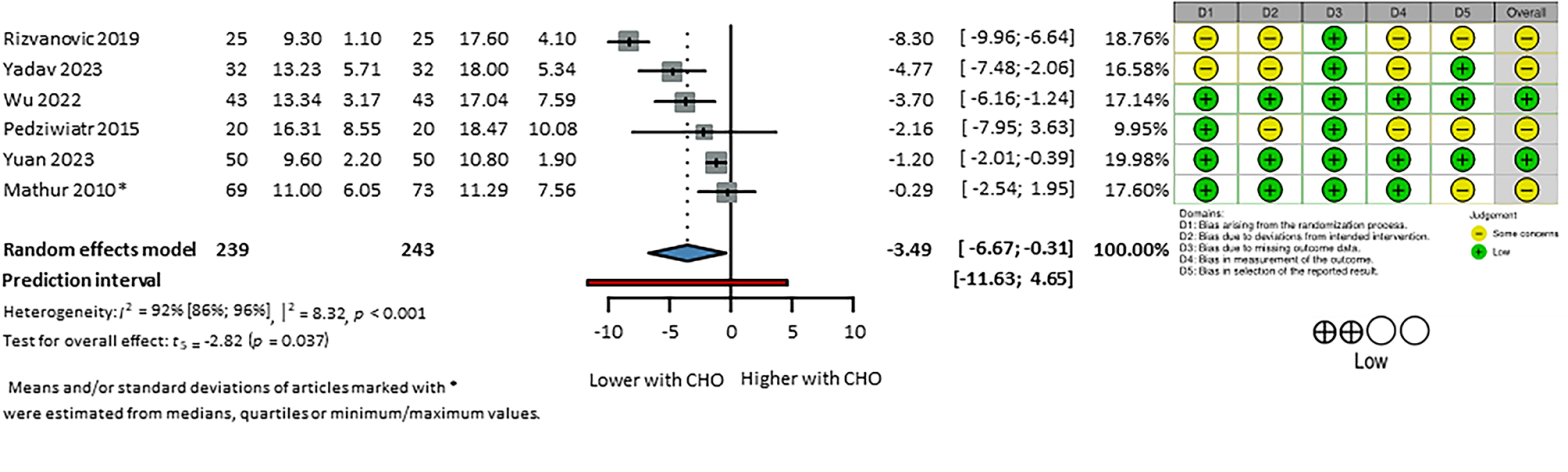
Serum insulin level on Postoperative Day 1 (CHO vs. No CHO—fasting or placebo). CHO; preoperative carbohydrate loading;. Risk of bias assessment with RoB2 tool displayed next to the respective article where a green (+) refers to low, and a yellow (−) refers to moderate risk of bias. D1: bias arising from randomization process, D2: bias due to deviations from intended interventions, D3: bias due to missing outcome data, D4: bias in measurement of the outcome, D5: bias in selection of the reported result. Summary of the level of certainty of evidence assessment by GRADE assessment on the bottom left corner.

Six studies^36,39,42,52,55,59^ including 482 patients reported the serum insulin levels on the postoperative day 1 (Fig. 4). These values were pre-prandial and were reported after the first 24-h period postoperatively, and they were measured alongside the serum glucose levels. The unit of measurement used to report the pooled estimates was IU/L, and results of studies reported using other units were converted to IU/L. The serum insulin levels on postoperative day 1 was significantly lower in the CHO and No-CHO groups (MD: − 3.49 [95% CI: − 6.67, − 0.31]). Results from the meta-analysis of serum insulin levels measured immediately after the operation are presented at Supplementary Fig. 4. There is no statistically or clinically significant difference between CHO and No-CHO groups: MD: − 3.0 [95% CI: − 1.49, 0.25].

### Postoperative CRP levels

We also investigated the effect of preoperative carbohydrate loading on the postoperative CRP levels. Four studies^27,29,36,42^ with 374 patients reported this outcome. There was no statistically or clinically significant difference between the CHO and No-CHO groups (MD: − 14.70 [95% CI: − 53.12, 23.71]) (See Supplementary Fig. 5.).

### Safety of preoperative carbohydrate loading

We also reviewed all of the included papers for systematic reporting of postoperative complications. Two studies reported such outcomes of interest (Table 2). It is important to note that while Gianotti et al.^23^ report a significantly increased incidence of postoperative diarrhea and vomiting, Rizvanovic et al.^43^ report a significant reduction in serious postoperative complications according to Clavien–Dindo Classification. Furthermore, similarly to the systematic reporting of Gianotti et al., aspiration was almost non-existent as a complication in either CHO or No-CHO groups in the included studies.

**Table 2:** Complications and mortality.

	Adverse event	CHO group	No-CHO group	*P*-value
Gianotti 2018	Diarrhea	26 (7.9%)	10 (3.0%)	0.009
	Abdominal distension	3 (0.9%)	0 (0%)	0.009
	Nausea	5 (1.5%)	2 (0.6%)	0.24
	Vomiting	17 (5.1%)	5 (1.5%)	0.009
	Aspiration episodes	0 (0%)	0 (0%)	1
Rizvanovic 2023	Total complications	16.70%	43.30%	
	Mortality	0%	1.70%	0.313
	Grade I*	10%	36.70%	0.015
	Grade II*	6.70%	50%	0.002
	Grade III*	0%	16.70%	0.01
	Grade IV*	0%	16.70%	0.01

*Clavien–Dindo

### Risk of bias and level of evidence certainty assessments

The overall risk of bias was moderate for nearly all included studies, despite the fact that they were all randomized controlled trials. In most cases, the lack of blinding was the major cause of risk of bias, in which case, it may be assumed that a bias may lead investigators to overestimate the beneficial effects of the intervention. This concern may be further highlighted by instances of possible deviations from the intended interventions.

The level of evidence certainty was assessed to be low overall. This is due to the risk of bias and imprecision of the results pooled from the articles included. The comprehensive summary of both risk of bias and level of evidence certainty assessments may be found in Supplementary Figs. 5 and 6, and Supplementary Table 1 respectively.

## Discussion

### Length of hospital stay

We considered the LOS as our primary outcome, as in our opinion this may represent the overall effects of preoperative carbohydrate loading best, due to the potential beneficial effects on several important confounders such as better postoperative blood sugar control, faster gastro-intestinal recovery, lesser chance of nosocomial infections and also the reduction of hospital costs^60^. Other safety outcomes were not assessed in this study.

We found that LOS of those patients who received carbohydrate loading was significantly shorter compared to preoperative fasting, and that is also a clinically relevant difference. There are several hypotheses regarding the underlying cause of earlier hospital discharge, but so far, no large-scale study such as a registry analysis has been conducted to answer this question.

Hospital LOS is influenced by multiple factors, such as adequate pain and fluid management, early mobilization, earlier start of oral food and fluid intake, etc., not analyzed in the current meta-analysis as data was either scarce or not reported at all. It is also important to note that there is a difference in the terms of ‘length of hospital stay’, ‘total lengths of hospital stay’ and ‘readiness to discharge’ that was also inconsistently defined in the articles. LOS may also be affected by local organizational issues such as timing of surgery after hospital admission and the timing of patient discharge. Therefore, we entirely agree with Cho et al.^19^, who suggested using the term, “time to ready to discharge” in the future as an outcome measure instead of the actual LOS.

Another methodological issue was that the use of placebo differed greatly in the studies. In some cases, the authors used plain water, while others used fluid with minerals that was not flavored, so proper blinding cannot be guaranteed which is a biasing factor all in general. Nevertheless, it is the opinion of the study authors, that a standardized supplementation most closely resembling the intervention in taste and feel is the most suitable placebo for investigating the effects of carbohydrate loading in a placebo-controlled trial design.

### Postoperative blood sugar and insulin levels

Postoperative hypo- and hyperglycemia should be avoided due to several potential adverse effects both surgical a general that may delay recovery in general after surgery^2^. In the current study we found that the postoperative metabolic status as indicated by serum glucose and insulin levels did not differ importantly between the groups, except for the insulin levels on the first postoperative day.

It is important to note that the amount used, composition, and timing of administration of CHO-loading fluids notably differed in the studies included. Another reason for heterogeneity is that several studies used different timepoints for measuring insulin and glucose levels. Some even recorded these parameters relatively late in the postoperative period, up to a week. It would be difficult to show the impact of such a relatively low-dose carbohydrate loading on the metabolic status after a week. Even measuring blood sugar levels on day 1 and 2 is questionable, especially if patients are fed already on day one. As for future research, we believe that timing of postoperative glucose and insulin level measurements should be standardized.

### C-reactive protein

CRP is the most commonly used inflammatory biomarker that may also be used for assessing postoperative inflammatory response^60^. However, evidence from current literature does not provide evidence to such an effect. How carbohydrate loading could affect postoperative inflammatory response remains uncertain and detailed description of the current hypotheses are beyond the scope of this manuscript.

### Strengths and limitations

The main strength of our analysis is that we strictly followed our protocol, which was registered in advance. Rigorous methodology was applied, and our results were derived from randomized controlled trials only. To our knowledge, the current study is the largest and most up-to-date on the subject of preoperative carbohydrate loading in major non-cardiac surgeries in general anesthesia.

The major limitation of the current study is the high heterogeneity of not only the data, but the setting of the investigations in the literature. We found very heterogeneity both between- and within-study assessments of the patient population. The definition of fasting and placebo also differed to some extent among the studies. The administered carbohydrate fluids varied in contents and amount. All limitations mentioned above limit the generalizability of our findings.

### Implication for practice and research

In practice, there remains little uncertainty regarding the feasibility and the safety of preoperative carbohydrate loading. What measurable effect this intervention has seems to be uniformly pointing towards non-inferiority, if not superiority. Finally, considering the potential for patient benefits not mentioned as a specific intentional effect of preoperative carbohydrate loading, such as lowering preoperative anxiety, we recommend widespread utilization of this intervention. Thus, the current study supports the ERAS guidelines’ recommendations for implementing preoperative carbohydrate loading in major non-cardiac surgery.

Based on the findings of the current study, the need for further research to discover the intervention modality and the patient population who benefit the most from this intervention remains an important task for future clinical researchers. In any and all future research on the subject, we suggest the using the term “readiness to discharge” instead of “length of hospital stay” as some confounding circumstances (such as social background, logistics, hospital bed availability), may be adding to the uncertainty we observed. Finally, we recognize that postoperative complications are rarely used as an outcome that should also be included as important endpoints on future trials. We would like to highlight the importance of implementing scientific results into daily practice^61,62^, according to which this approach may allow faster recovery and lesser incidence of postoperative complications.

## Conclusion

Preoperative carbohydrate loading was found to reduce LOS compared to fasting or placebo. This finding should encourage clinicians to comply with the ERAS recommendations, and future researchers to optimize the intervention for the best possible benefit.

## Electronic supplementary material

Below is the link to the electronic supplementary material.

Supplementary Material 1

## Data Availability

The datasets used and/or analysed during the current study available from the corresponding author on reasonable request.
