# Supplementary material for: Preoperative carbohydrate loading reduces length of stay after major elective, non-cardiac surgery when compared to fasting: a systematic review and meta-analysis
Source: Sci Rep. 2025 May 31;15:19119. doi: 10.1038/s41598-025-00767-z (PMC12126546; doi:10.1038/s41598-025-00767-z)
Supplement: Supplementary file 1 — Supplementary Material 1 [file 41598_2025_767_MOESM1_ESM.zip › Baseline supplementary.docx]

| **Author Year** | **Intervention group** | | | | | | | **Comparator/Control group** | | | | | | |
| --- | --- | --- | --- | --- | --- | --- | --- | --- | --- | --- | --- | --- | --- | --- |
|  | **Number of patients** | **Age** | **Sex (female% of total)** | **ASA** | | | **BMI** | **Number of patients** | **Age (years)** | **Sex (female% of total)** | **ASA** | | | **BMI** |
|  |  |  |  | **I** | **II** | **III** |  |  |  |  | **I** | **II** | **III** |  |
| Cakar 2017 | 30 | 48.17 ± 9.81 years | 60% | 0 | 26 | 4 | 25.09 ± 3.37 | 30 | 50.07 ± 9.95 years |  | 0 | 28 | 2 | 26.33 ± 3.43 kg/m² |
| Cho 2021 | 32 | 40 ± 12 years | 100% | 27 | 5 | 0 | na | 32 | 38 ± 10 years | 100% | 25 | 7 | 0 | na |
| Cho 2021* | 44 | 41.3 ± 11.9 years | 100% | 34 | 10 | 0 | 41.3 ± 11.9 years | 44 | 38.9 ± 10.9 years | 100% | 35 | 9 | 0 | 38.9 ± 10.9 years |
| Deng 2022 | 40 | 55.25 ± 13.45 years, | 35% | 8 | 31 | 1 | 23.12 ± 3.35 kg/m² | 42 | 55.81 ± 13.83 years | 50% | 11 | 30 | 1 | 55.81 ± 13.83 years |
| Dilmen 2017 | 20 | 48.95 ± 11.51 years | 50% | 13 | 7 | 0 | 26.57 ± 1.18 kg/m² | 20 | 45.25 ± 7.23 years | 50% | 12 | 8 | 0 | 45.25 ± 7.23 years |
| Faria 2009 | 11 | 47 years (range 19–65) | 100% | 5 | 6 | 0 | na | 10 | 48 years (range 29–65) | 100% | 4 | 6 | 0 | na |
| Gianotti 2018 | 331 | 68.0 years (IQR: 58.0; 75.5) | 42.90% | 25 | 212 | 93 | 25.5 (IQR: 23.11; 27.47) | 331 | 67.0 years (IQR: 57.0; 75.0) | 65.90% | 24 | 217 | 83 | 25.2 (IQR: 23.12; 27.68) |
| Gümüs 2021 | 35 | 48.68 ± 9.61 years | 71.40% | na | na | na | Normal (14.3%), Overweight (45.7%), Class I obese (20.0%), Class II obese (20.0%) | 33 | 42.38 ± 9.27 years | 69.60% | na | na | na | Normal (27.2%), Overweight (30.3%), Class I obese (36.2%), Class II obese (6.06%) |
| Hamamoto 2018 | 31 | 71 (range: 43–84) years | 45% | 8 | 23 | 0 | 21.3 (14.7–32.8) kg/m² | 33 | 69 (range: 49–85) years | 52% | 6 | 27 | 0 | 21.5 (16.7–26.2) kg/m² |
| Hausel 2005 | 55 | mean: 48.3 years | 74.5 | na | na | 0 | 24.2 kg/m² | 59 (placebo group) | mean: 46.8 years | 69.50% | na | na | 0 | 23.8 kg/m² |
| Henriksen 2003 | 17 | mean: 64 (range: 44-82) | na | na | na | na | mean: 27 (range: 20-35) | 16 | mean: 64 (range: 52-81) | 50% | na | na | na | mean: 26 (range: 21-33) |
| Hu 2021 | 28 | 71.7 (IQR: 68.5–74.5) | na | 5 | 17 | 6 | 23.54 (IQR: 22.1–26.3) | 30 | 70.5(IQR: 68.5–75.0) | na | 6 | 18 | 6 | 23.86 (IQR: 21.4–26.28) |
| Kaska 2010 | 74 | na | na | na | na | na | na | 75 | na | na | na | na | na | na |
| Kumar 2024 | 36 | 56.60 ± 13.10 years | 61.20% | na | na | na | 23.92 ± 2.49 | 36 | 52.20 ± 13.90 years | 52.80% | na | na | na | 23.92 ± 2.49 |
| Lai 2024 | 30 | 65 ± 7.8 years | 80% | 0 | 3 | 27 | 26.1 ± 3.7 | 30 | 67 ± 7.9 years | 70% | 0 | 2 | 28 | 27.4 ± 2.3 |
| Lee 2018 | 46 | 50 ± 13 years | 50% | na | na | 0 | 24.5 ± 4.8 kg/m² | 51 | 49 ± 12 years | 50% | na | na | 0 | 25.4 ± 4.0 kg/m² |
| Li 2022 | 31 | 62 (IQR: 55 - 66) | 29% | 0 | 23 | 8 | 25.4 ± 2.7 kg/m² | 32 | 68 (IQR: 55-66) | 44% | 0 | 21 | 11 | 25.2 ± 5.1 kg/m² |
| Lin 2022 | 19 | 62.1 ± 10.9 years | 37% | 0 | 17 | 2 | 25.3 ± 3.1 kg/m² | 19 | 62.0 ± 8.8 years | 40% | 0 | 18 | 2 | 23.8 ± 2.3 kg/m² |
| Liu 2019 | 58 | na | 70.7 | 8 | 50 | na | na | 62 | na | 64.5 | 13 | 49 | 0 | na |
| Mathur 2023 | 45 | na | na | na | na | na | na | 45 | na | na | na | na | na | na |
| Mathur 2010 | 69 | na | na | 9 | 42 | 18 | na | 73 | na | 40 | 10 | 51 | 12 | na |
| Noblett 2006 | 12 | 58 | na | na | na | na | na | 24 | 57 | na | na | na | na | na |
| Onalan 2019 | 25 | 53-16 | 80 | na | na | na | 28.3 ± 3.7 | 25 | 54-14 | 68 | na | na | na | 29.0 ± 3.3 |
| Pedziwiatr 2015 | 20 | 53.27+15.29 | 60 | 6 | 13 | 1 | 28.78 | 20 | 55+9.55 | 65 | 3 | 14 | 3 | 28.76 |
| Qin 2022 | 114 | 58.39 ± 7.55 | 36 | na | na | na | na | 117 | 59.05 ± 7.65 | 46.2 | na | na | na | na |
| Rajan 2021 | 26 | 47.5 ± 13.9 | 73.1 | 15 | 11 | na | na | 26 | 15.6 ± 13.0 | 84.6 | 7 | 19 | 0 | na |
| Rizvanovic 2019 | 25 | 61.0 ± 7.3 | 44 | 8 | 17 |  | 26.4 ± 4.5 | 25 | 60.2 ± 9.7 | 48 | 7 | 18 | 0 | 24.7 ± 1.6 |
| Rizvanovic 2023 | 30 | 60.90 ± 6.80 | 43.3 | 8 | 11 | 11 | 23.56 ± 1.51 | 30 | 59.93 ± 9.31 | 46.7 | 9 | 10 | 11 | 24.70 ± 1.68 |
| Sada 2014 | 44 | 56.9 ± 12.75 | 61.4 | na | na | na |  | 96 | 56.08 ± 14.0 | 64.6 | na | na | na | na |
| Senapathi 2022 | 34 | 44.12+10.73 | 20.6 | na | na | na | 21.61 ± 2.3 | 34 | 44.88+10.98 | 29.4 | na | na | na | 20.23 ± 1.73 |
| Shi 2020 | 21 | 55 (36-74) | 47.6 | 3 | 18 |  | 23 (20-25) | 42 | 55.5 (40-72) | 47.6 | 7 | 35 | 0 | 22.5 (18-26) |
| Sio 2015 | 71 | na | na | na | na | na | na | 71 | na | na | na | na | na | na |
| Tavalaee 2022 | 50 | na | na | na | na | na | na | 45 | na | na | na | na | na | na |
| Varughese 2024 | 25 | 59.08 ± 10.25 | 60 | na | na | na | 27 ± 4.92 | 25 | 58.7 ± 8.29 | 44 | na | na | 0 | na |
| Wang 2019 | 36 | 57.50±2.30 | 36.1 | 7 | 29 | na | na | 37 | 55.59±2.10 | 35.1 | 5 | 32 | 0 | na |
| Wang 2024 | 84 | 47.7 ± 12.2 | 73.8 | 70 | 14 | na | 25 | 45 | 49.0 ± 12.3 | 55.6 | 36 | 9 | 0 | 24.7 |
| Wu 2022 | 43 | 64.1 ± 6.0 | 39.5 | 3 | 38 | 2 | 22.82 ± 2.87 | 43 | 62.7 ± 6.3 | 32.6 | 6 | 34 | 3 | 23.07 ± 2.89 |
| Yadav 2023 | 32 | 42.53 ± 8.88 |  | na | na | na | 24.23 | 32 | 43.63 ± 8.41 |  | na | na | na | 24.22 |
| Yang 2012 | 24 | 63.38 ± 9.07 | 29.17 | 8 | 12 | 4 | 23.64 ± 2.80 | 24 | 62.58 ± 10.22 | 29.17 | 9 | 12 | 3 | 22.91 ± 3.19 |
| Yuan 2023 | 50 | 53.1 ± 8.2 | 48 | 22 | 28 | 0 | 25.2 ± 4 | 50 | 52.3 ± 9.2 | 44 | 24 | 26 | 0 | 25.3 ± 3.9 |
| Yuill 2005 | 31 | 52.8 ± 2.5 | 35.5 | na | na | na | 25.2 ± 1.2 | 34 | 52.1 ± 2.4 | 44.1 | na | na | na | 25.1 ± 1.7 |
| Zhang 2022 | 100 | 45.8 ± 10 | 100 | 81 | 19 | 0 | 22.9 ± 2.55 | 100 | 45.6 ± 9 | 100 | 82 | 18 | 0 | 23.9 ± 3.4 |
