# Supplementary material for: Preoperative carbohydrate loading reduces length of stay after major elective, non-cardiac surgery when compared to fasting: a systematic review and meta-analysis
Source: Sci Rep. 2025 May 31;15:19119. doi: 10.1038/s41598-025-00767-z (PMC12126546; doi:10.1038/s41598-025-00767-z)

**Supplementary Material**

**Supplementary Table 1:** GRADE assessment

| **Certainty assessment** | | | | | | | **№ of patients** | | **Effect** | | **Certainty** | **Importance** |
| --- | --- | --- | --- | --- | --- | --- | --- | --- | --- | --- | --- | --- |
| **№ of studies** | **Study design** | **Risk of bias** | **Inconsistency** | **Indirectness** | **Imprecision** | **Other considerations** | **Oral CHO** | **Placebo or fasting** | **Relative (95% CI)** | **Absolute (95% CI)** |  |  |
| **Length of stay** | | | | | | | | | | | | |
| 23 | randomised trials | not serious | serious^a^ | not serious | serious^b^ | none | 1293 | 1316 | - | MD **0.56 days fewer** (2.61 fewer to 1.49 more) | ⨁⨁◯◯ Low^a,b^ |  |
| **Postoperative blood glucose (follow-up: 1 days)** | | | | | | | | | | | | |
| 13 | randomised trials | not serious | serious^a^ | not serious | serious^b^ | none | 907 | 919 | - | MD **0.67 mg/dl lower** (1.23 lower to 0.11 lower) | ⨁⨁◯◯ Low^a,b^ |  |
| **Postoperative insulin (follow-up: 1 days)** | | | | | | | | | | | | |
| 6 | randomised trials | not serious | serious^a^ | not serious | serious^b^ | none | 239 | 243 | - | MD **3.49 uU/ml lower** (6.67 lower to 0.31 lower) | ⨁⨁◯◯ Low^a,b^ |  |

**CI:** confidence interval; **MD:** mean difference

**Explanations**

a. Significant heterogeneity

b. Confidence interval includes significant benefit and no effect

**Supplementary Figure 1:** Postoperative CRP Day 1


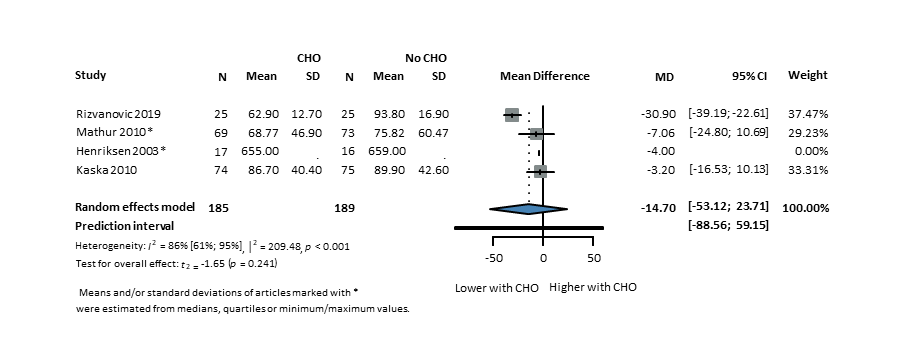


**Supplementary Figure 2:** Postoperative blood glucose (immediate)


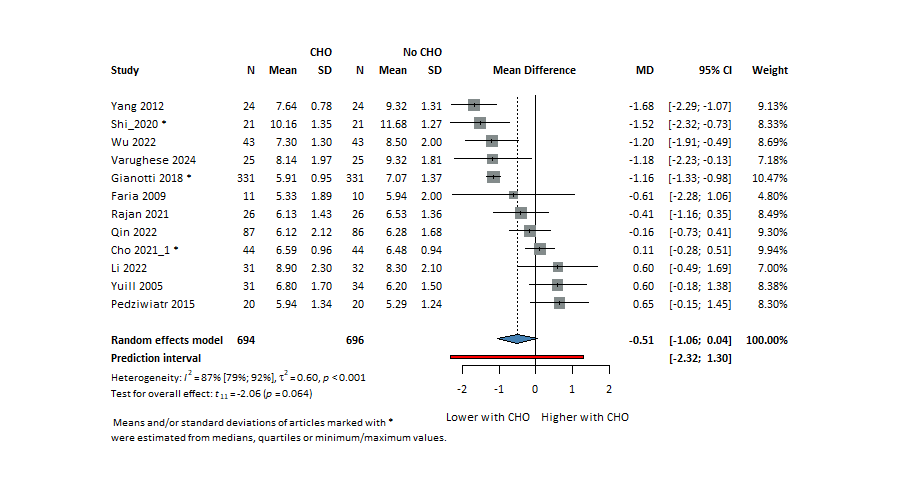


**Supplementary Figure 3:** Postoperative glucose (earliest)


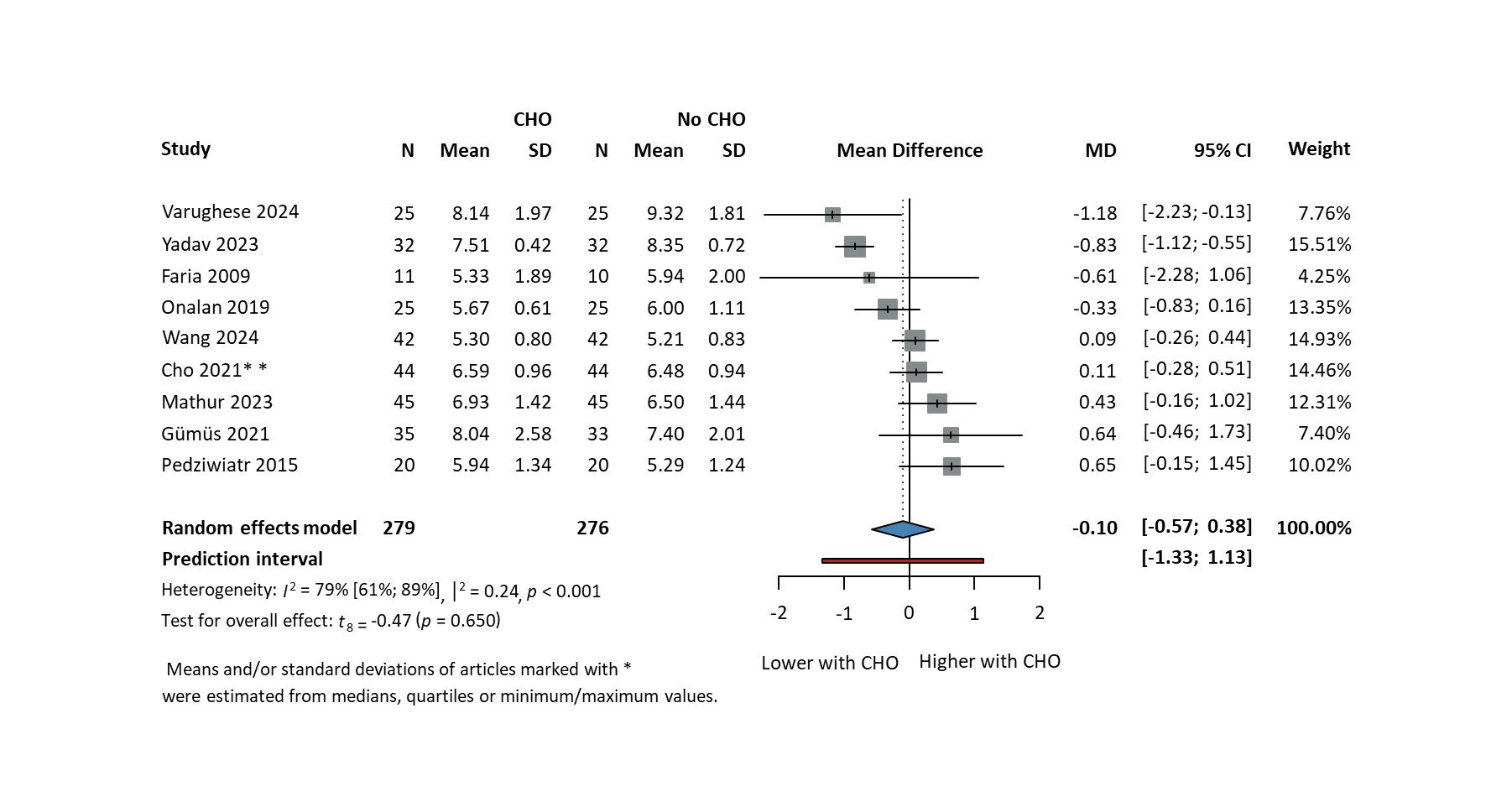


**Supplementary Figure 4:** Postoperative insulin (immediate)


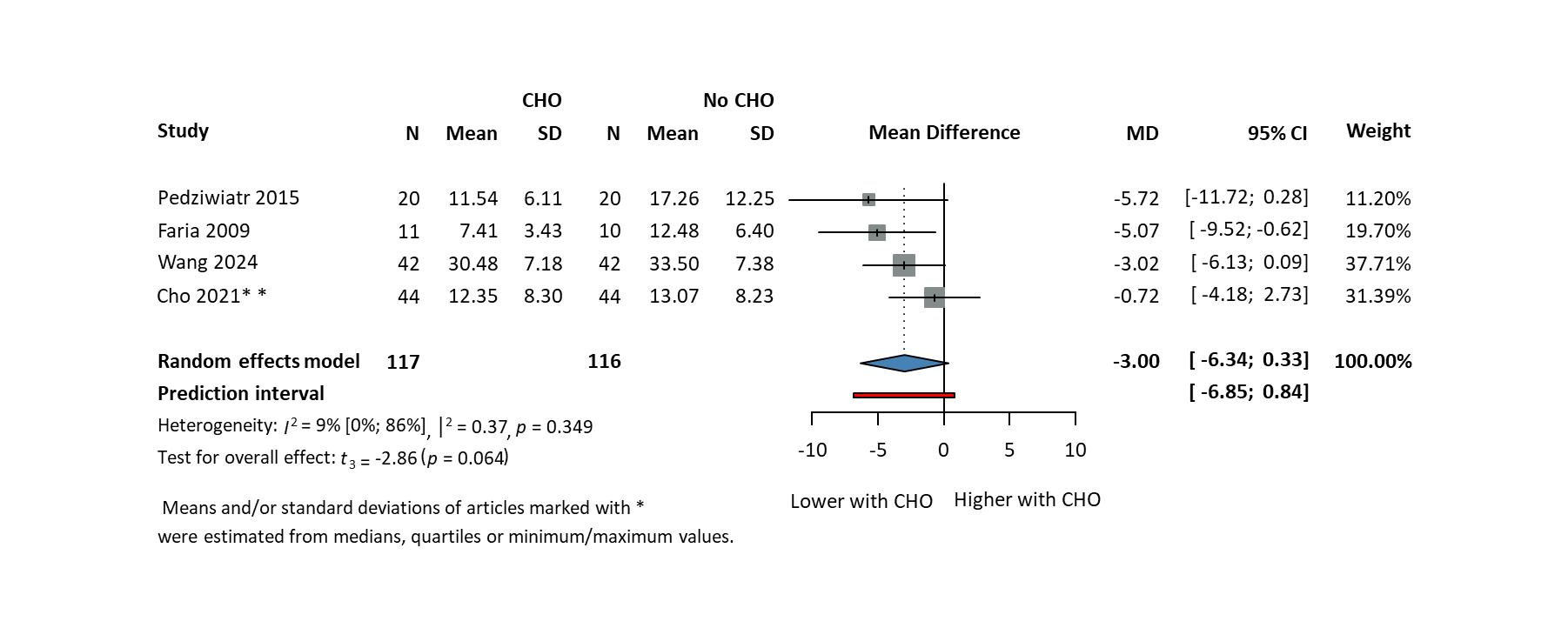


**Supplementary Figure 5:** Summary of RoB of all papers


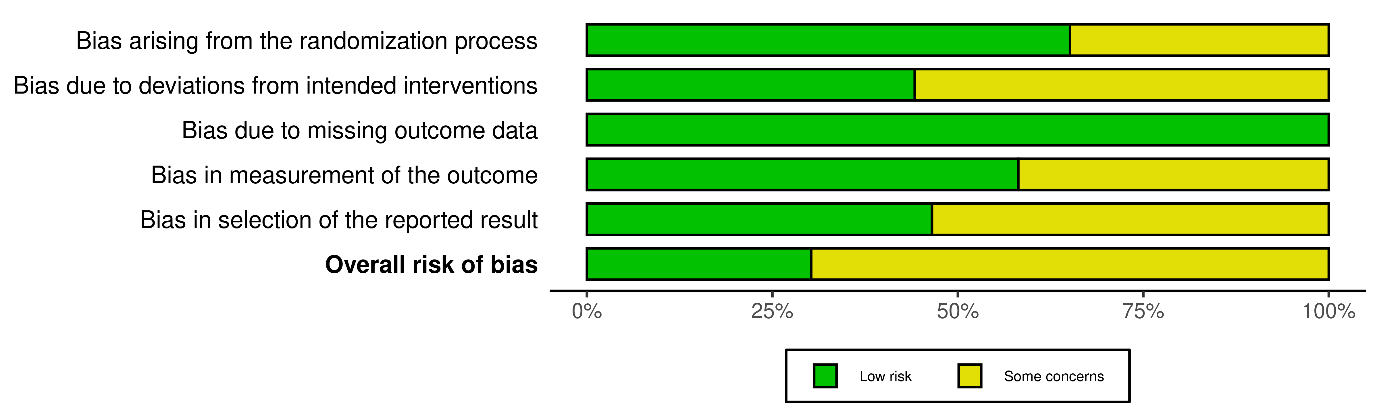


**Supplementary Figure 6:** RoB of all papers


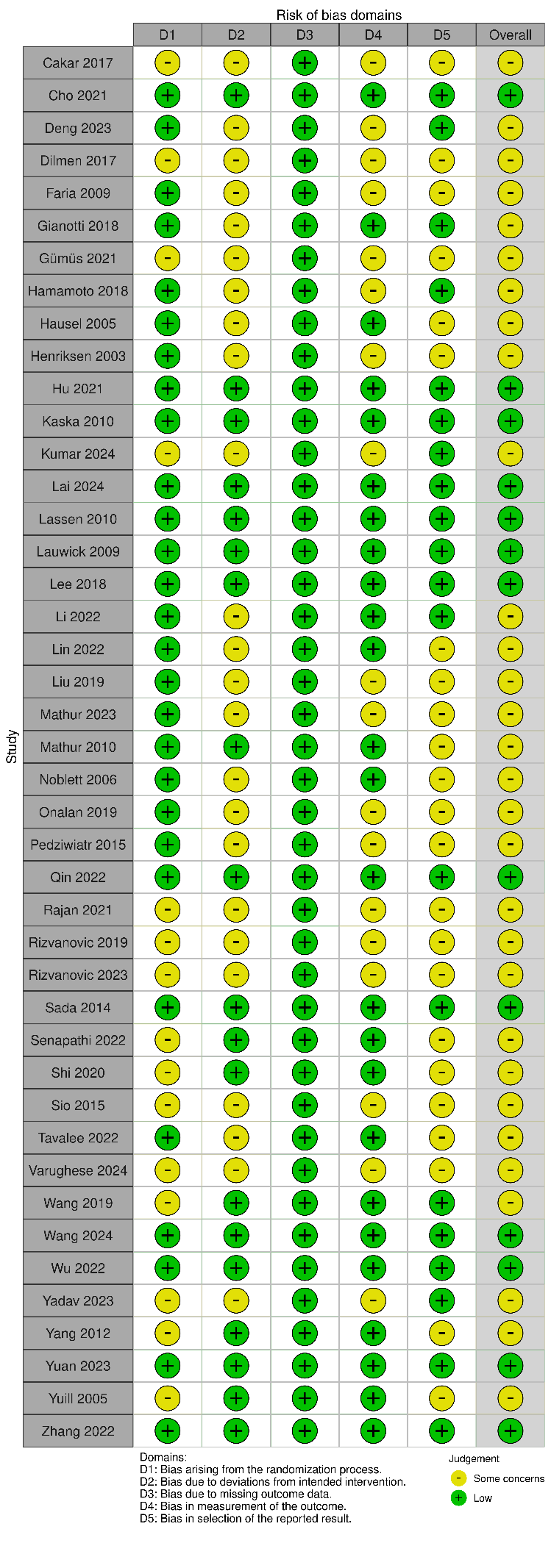

Supplement: Supplementary file 1 — Supplementary Material 1 [file 41598_2025_767_MOESM1_ESM.zip › Supplement.docx]
